# Application of POSSUM and P-POSSUM scores in the risk assessment of elderly hip fracture surgery: systematic review and meta-analysis

**DOI:** 10.1186/s13018-022-03134-0

**Published:** 2022-05-07

**Authors:** Feng Wanjiang, Zhang Xiaobo, Wu Xin, Meng Ye, Huang Lihua, Wang Jianlong

**Affiliations:** 1 Department of Orthopedics, The Third Xiangya Hospital of Central South University, Changsha, Hunan China; 2 Center for Experimental Medicine, Third Xiangya Hospital of Central South University, No.138 Tongzipo Road, Changsha, 410013 Hunan China

**Keywords:** POSSUM, P-POSSUM, Hip fracture, Morbidity, Mortality, Meta-analysis

## Abstract

**Background:**

Since Mohamed et al. analyzed 2326 orthopedic cases in 2002 and believed that the POSSUM formula can be directly used to predict postoperative morbidity and mortality in orthopedic patients, applications of POSSUM and P-POSSUM scores in the hip fracture surgery have been mostly reported in the field of orthopedics, but there are still some inconsistencies in the related reports.

**Methods:**

The electronic library was searched for all literature that met the purpose from its inception to 2021. Relative risk (RR) was selected to evaluate whether the model could be used to assess the risk of surgery in patients with elderly hip fractures. Finally, sensitivity analyses and subgroup analyses were performed.

**Results:**

Thirteen studies were finally included, including 9 retrospective and 4 prospective studies.The morbidity analysis includes 11 studies, and the result was RR = 1.07 (95% CI 0.93–1.24), The mortality analysis includes 11 studies on POSSUM and 5 studies on P-POSSUM. The results of mortality by POSSUM and by P-POSSUM were RR = 1.93 (95% CI 1.21–3.08) and RR = 1.15 (95% CI 0.89–1.50), respectively. POSSUM had more accuracy to predict mortality for sample < 200 subgroup(RR = 2.45; 95% CI 0.71–8.42) than sample > 200 subgroup(RR = 1.59; 95% CI 1.06–2.40), and in the subgroup of hip fractures that did not distinguish between specific fracture types(RR = 1.69, 95% CI 0.87–3.32) than intertrochanteric neck fracture subgroup(RR = 5.04, 95% CI 1.07–23.75) and femoral femoral fracture subgroup(RR = 1.43,95% CI 1.10–1.84).

**Conclusion:**

POSSUM can be used to predict morbidity in elderly hip fractures. The P-POSSUM was more accurate in predicting mortality in elderly hip fracture patients compared to the POSSUM, whose predictive value for mortality was influenced by the sample size and type of fracture studied. In addition, we believe that appropriate improvements to the POSSUM system are needed to address the characteristics of orthopedic surgery.

## Introduction

Hip fractures mainly refer to femoral neck fractures and intertrochanteric fractures. Gullberg et al. [1] predict that the global number of hip fractures will reach 2.6 million and 4.5 million by 2025 and 2050. With the aging of the global population, the incidence of elderly hip fractures in developed countries can reach 350/100,000[2], becoming one of the most common diseases in joint orthopedics. Generally, the main treatment for hip fractures is surgery, however elderly patients with hip fractures often have many basic diseases when they are admitted to the hospital. George et al. [3] found that when elderly patients with hip fractures had a variety of other diseases, the risk of postoperative morbidity and mortality increased. Their data showed that 20% of the patients had postoperative complications, and the 30-day death rate of such patients was 9%. In addition, previous studies have also shown that the ageing of patients, cognitive dysfunction and other body aging processes will also increase the risk of postoperative complications or death in patients with hip fractures [4–6]. In actual clinical work, active preoperative preparation will significantly reduce the adverse events of patients during the perioperative period. Surgical risk prediction models are a very valuable tool for surgeons because these tools allow surgeons to assess and prevent patients’ perioperative events in advance, so as to make the best decisions to optimize resources and improve the quality of care for patients [7, 8].

Many prediction models have emerged in recent years, but their specific application value is almost limited. The Physiological and Operative Severity *Score* for enUmeration of mortality and morbidity (POSSUM) was first proposed by Copeland et al. [9] in 1991 to evaluate the prognosis of patients. Subsequently, Whiteley et al. [10] found that the POSSUM scoring system would overestimate the postoperative mortality rate, and after simplifying the exponential analysis technology in the POSSUM scoring system, a P-POSSUM scoring formula that was more suitable for surgical patients was obtained. Currently, the APACHEI, APACHE II, POSSUM, ASA and NHFS scoring systems are generally used internationally to assess the perioperative risk of surgical patients, and the research by de Cássia Braga Ribeiro et al. [11] believes that the POSSUM scoring system has the most application value in the risk assessment of the perioperative period. Some scholars believe that the Surgical Risk Scale (SRS) is more accurate than the POSSUM scoring system for surgical risk assessment, has the advantages of simple procedures, and can obtain the predictive indicators before surgery [12], but in disciplines of general surgery, vascular surgery and esophageal surgery, the POSSUM scoring system is still widely used and recognized by surgeons [13–17].

Since Mohamed et al. [18] analyzed 2326 orthopedic cases in 2002 and believed that the POSSUM formula can be directly used to predict postoperative complications and death risks in orthopedic patients, more and more reports have appeared on the application of POSSUM and P-POSSUM scores in hip fracture surgery. Yet, there were inconsistencies in the related reports, some reports believed that the POSSUM scoring system could not accurately predict postoperative morbidity and mortality, but other reports supported this model [19–22]. Therefore, the purpose of this study is to conduct a meta-analysis on the application value of POSSUM and P-POSSUM in the risk assessment of hip fracture surgery, in order to guide the orthopedic surgeon in the evaluation of postoperative risk events and the choice of surgical benefits.

## Methods

### Search strategy

The search-style electronic libraries, including Pubmed, Embase, the Cochrane Library, CNKI, Wanfang Data, VIP Chinese Journals, and China Biomedical Literature Service System were used for document retrieval. From the establishment of the databases to 2021, a total of 289 documents were retrieved. All documents had abstracts or full texts, and there are no language restrictions. We used POSSUM or P-POSSUM combined with fractures for literature search [Pubmed's search formula: (("Fractures, Bone"[Mesh]) OR(((((((((((((fracture[Title/Abstract]) OR (Broken Bones[Title/Abstract])) OR (Bone,Broken[Title/Abstract])) OR (Bones, Broken[Title/Abstract])) OR (Broken Bone[Title/Abstract])) OR (Bone Fractures[Title/Abstract])) OR (Bone Fracture[Title/Abstract])) OR (Fracture,Bone[Title/Abstract])) OR (Spiral Fractures[Title/Abstract])) OR (Fracture,Spiral[Title/Abstract])) OR (Fractures, Spiral[Title/Abstract])) OR (SpiralFracture[Title/Abstract])) OR (Torsion Fractures[Title/Abstract])) OR (Fracture,Torsion[Title/Abstract])) OR (Fractures, Torsion[Title/Abstract])) OR (TorsionFracture[Title/Abstract]))) AND( (POSSUM[Title/Abstract]) OR (P-POSSUM[Title/Abstract]))]. After eliminating the duplicate documents, all the review documents retrieved by this retrieval method and the references of the original research were comprehensively reviewed to determine whether there were additional documents. EndNote X9 software was used to manage the documents.

### Inclusion and exclusion criteria

Inclusion criteria: original retrospective or prospective cohort study; the literature using POSSUM or P-POSSUM for research; patients with bone fracture; surgical treatment. Exclusion criteria: meeting or review; graduate thesis; non-hip fractures (including femoral neck fractures and intertrochanteric fractures); data was incomplete (the predicted value and/or observed value cannot be obtained); Study population age < 60 years; There is no defined follow-up period (period of hospitalisation or number of days of follow-up).

Because there was no significant difference in composition between the orthopedic POSSUM system (O-POSSUM) modified by Mohamed et al. [18] based on the characteristics of orthopedic surgery in 2002 and the original POSSUM score proposed by Copeland et al. [9] in 1991, no strict distinction was made in literature inclusion.

### Literature review and data extraction

The titles and abstracts were used to screen the literature for inclusion criteria. After the data extraction, the NOS scale was used to evaluate the quality of the literature. A summary of the literature content is shown in Table 1, which is recorded in sequence with the following items: author name; publication year; country of author; study type; fracture type; total sample size; predictive outcome indicators (POSSUM: morbidity and mortality),P-POSSUM: mortality); actual outcome indicators (morbidity and mortality); O/E value (observed value/predicted value); NOS score result.

**Table 1:** Summary of included articles

Authors	Year	Country	Fracture type	Patient's age	Follow-up time	Sample size (cases)	Predictive					O/E^1^	O/E^2^	O/E^3^	NOS score results
							POSSUM		P-POSSUM	Observed					
							Morbidity (cases)	Mortality (cases)	Mortality (cases)	Morbidity (cases)	Mortality ( cases)				
Rananathan. et al	2005	England	Femoral neck	75Y > 86%	30d	1164	–	181	–	–	119	–	0.66	–	*.6
Wang et al	2008	China	Hip	60Y = 100%	30d	295	94	21	–	97	9	1.03	0.43	–	*.7
Liu et al	2009	China	Femoral neck	80Y = 100%	14d	78	32	–	–	24	–	0.75	–	–	*.7
Liu et al	2010	China	Intertrochante	75Y = 100%	30d	30	4	–	–	3	–	0.75	–	–	*.6
Liu et al	2010	China	Intertrochante	60Y = 100%	30d	119	42	11	–	39	5	0.93	0.45	–	*.7
Wu etal	2011	China	Hip	60Y = 100%	30d	191	93	24	7	80	3	0.86	0.13	0.43	*.6
Hapuarachchi et al	2014	New Zealand	Femoral neck	90Y = 100%	30d	146	83	23	–	81	21	0.98	0.91	–	*.*7*
Wang et al	2016	China	Hip	60Y–103Y	30d	654	–	84	30	–	25	–	0.30	0.83	*.7
Liu et al	2017	China	Hip	60Y–87Y	30d	92	31	8	3	27	1	0.87	0.13	0.33	*.6
Blay-Domfnguez et al	2018	Spain	Hip	65Y = 100%	1Y	229	132	35	–	77	38	0.58	1.09	–	*.7
M. H. Jonsson	2018	Sweden	Hip	76Y–90Y	30d	997	411	69	63	407	62	0.99	0.90	0.98	*.7
Zhou et al	2019	China	Intertrochante	80Y = 100%	30d	148	23	84	11	34	8	1.48	0.10	0.73	*.7
Zaki et al	2019	Egypt	Hip	60Y = 100%	6 m	97	50	3	–	64	18	1.28	6.00	–	*.*7*
							4240	995	543	114	933	309 (99#)	0.94	0.57	0.87

O/E^1^: O/E value of the accuracy of POSSUM predicting the postoperative complications in elderly patients with hip fractures. O/E^2^: O/E value of the accuracy of POSSUM predicting the postoperative deaths in elderly patients with hip fractures. O/E^3^: O/E value of the accuracy of P-POSSUM predicting the postoperative deaths in elderly patients with hip fractures. #: The case of deaths observed by P-POSSUM study Cohort. *: NOS score

### Statistical analysis

We performed meta-analysis using the latest version of RevMan 5.4 software recommended by the Cochrane Library. We used the relative risk (RR) to assess the predictive accuracy of the POSSUM scoring system because the included studies were cohort studies, the data type was a dichotomous variable and RR was the most useful indicator of the strength of the event association. 95% confidence intervals (CI) for the RR were used to indicate accuracy, and when the horizontal line of the 95% CI intersected the null vertical line or the 95% CI contained 1, we considered the POSSUM to accurately predict the postoperative risk of patients. When the 95% CI did not intersect the null vertical line or when the 95% CI did not contain 1. We considered the POSSUM to be too high (both upper and lower 95% CI were greater than 1) or too low (both upper and lower 95% CI were less than 1) to predict the postoperative risk. When the heterogeneity *I*^*2*^ > 50% in the forest plot, we considered significant heterogeneity and chose the random-effects model; otherwise, we considered little heterogeneity and chose the fixed-effects model. Sensitivity analysis was performed for each of the included studies when *I*^*2*^ > 50%, and if one study was excluded, *I*^*2*^ < 50% was considered as the main source of heterogeneity; if sensitivity analysis did not reveal the main source of heterogeneity, subgroup analysis was performed according to hip fracture type (femoral neck fracture, intertrochanteric fracture or hip fracture) and sample size (less than 200 or greater than or equal to 200) to explore sources of heterogeneity.

## Results

### Process of literature inclusion

**Fig. 1:**
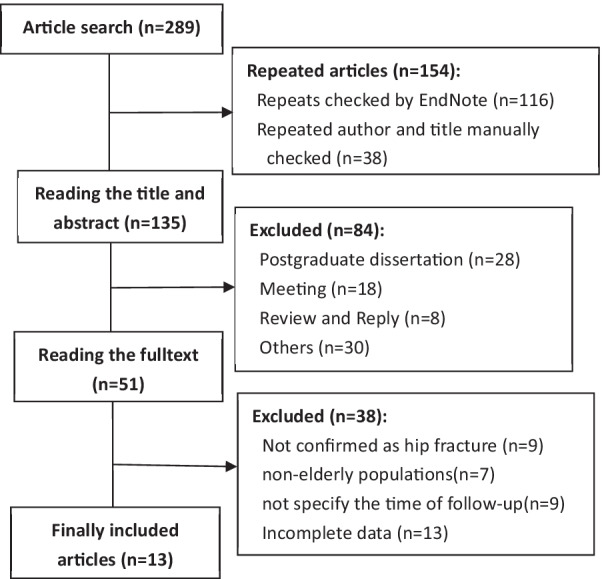
Process of inclusion and exclusion of articles

Total of 289 studies was obtained through a literature search, and the full text of 51 studies was obtained. Among them, 38 studies met the exclusion criteria: 9 studies could not determine whether it was a hip fracture; 7 studies were in non-elderly populations; 9 studies did not specify the duration of follow-up; 13 studies did not have complete data. In the end, 13 studies were used for meta-analysis [20–32], including 9 retrospective cohort studies [20, 22–26, 28, 29, 31] and 4 prospective cohort studies [21, 27, 30, 32]. The ratings of quality appraisal of these included papers are all ≥ 6 stars. The specific inclusion and exclusion process is shown in Fig. 1.

### Description of the meta-analysis results

A total of 13 studies were included, including 9 retrospective cohort studies and 4 prospective cohort studies. Among them, 11 studies each reported POSSUM's predictive value for the morbidity and mortality, and 5 studies reported the predicted value of P-POSSUM on the number of mortality. The total samples are 4240 patients with hip fractures, of which the prediction by POSSUM of number of postoperative morbidity in 2422 patients and number of postoperative mortality in 4312 patients, and the prediction by P-POSSUM of number of postoperative mortality in 2082 patients, are reported respectively. POSSUM predicted 995 postoperative morbidity, actually 933 postoperative morbidity were observed; POSSM and P-POSSUM predicted 543 and 114 postoperative mortality, respectively, actually 309 and 99 postoperative mortality were observed.

### The combined analysis results of POSSUM and P-POSSUM on the postoperative morbidity and mortality of patients with hip fracture

**Fig. 2:**
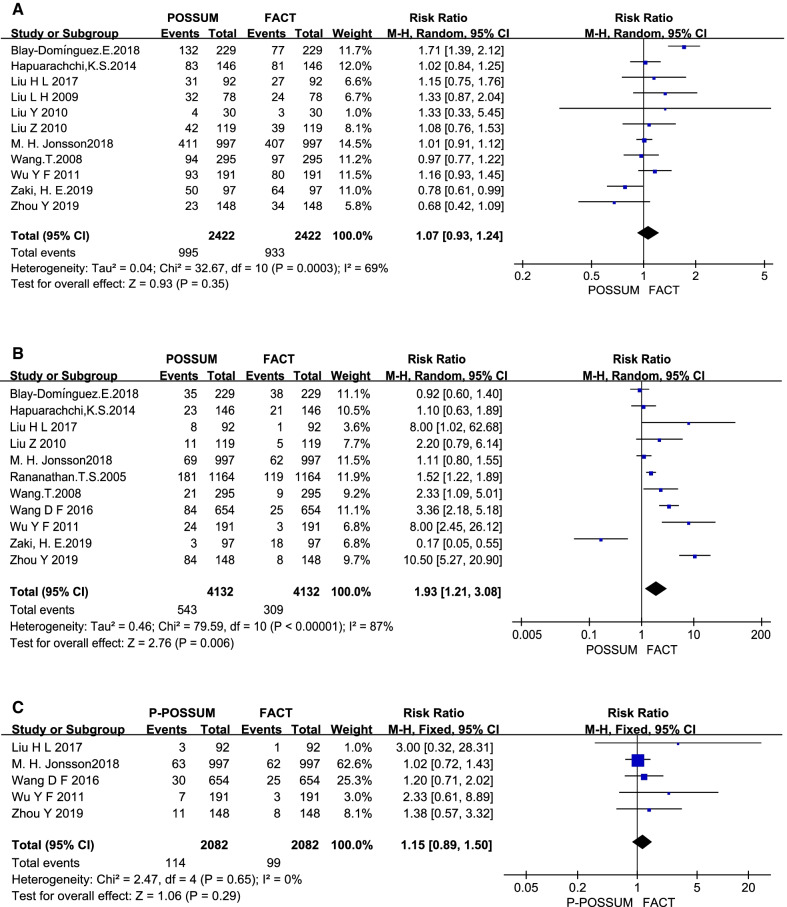
The accuracy of POSSUM or P-POSSUM predicting the postoperative complications or mortality in patients with hip fracture. (**A**) POSSUM for complications; (**B**) POSSUM for mortality; (**C**) P-POSSUM for mortality

**Fig. 3:**
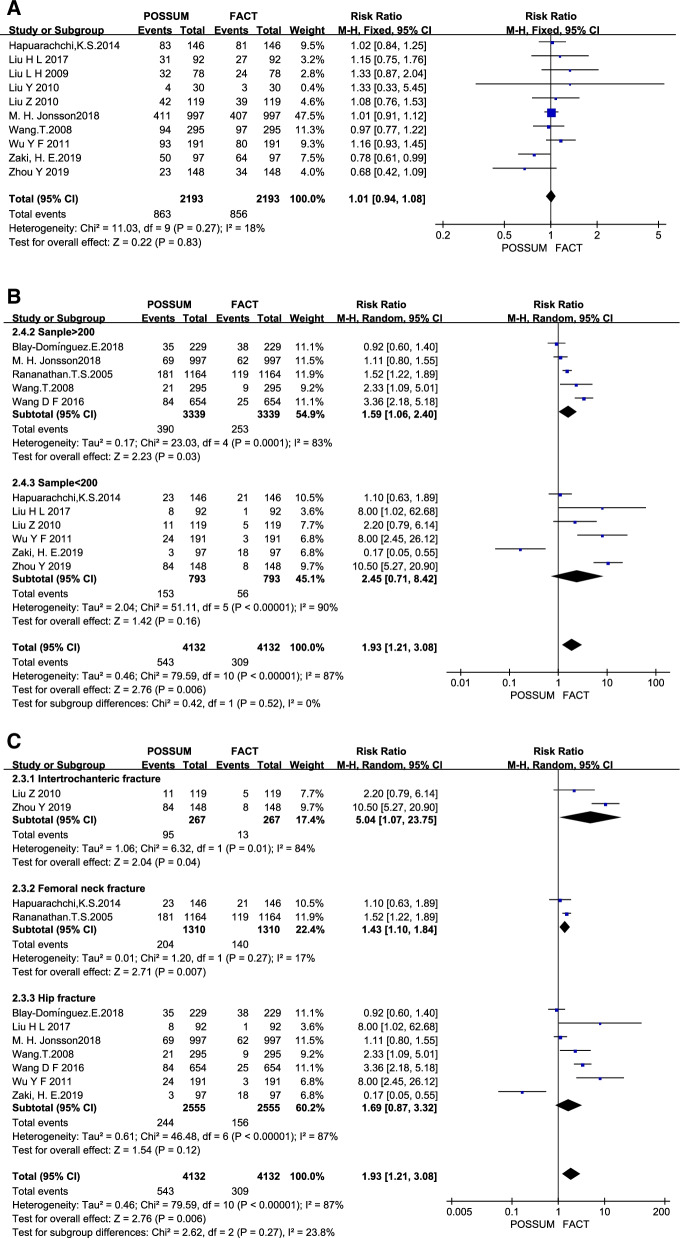
Heterogeneity analysis (**A**) Sensitivity analysis of POSSUM for the prediction of complications; (**B**) Sample size subgroup analysis of mortality prediction by POSSUM; (**C**) Fracture type subgroup analysis of mortality prediction by POSSUM

According to the meta results of the combined analysis, POSSUM accurately predicts the postoperative complications and over-predicts the postoperative mortality of hip fracture patients, while P-POSSUM can accurately predict the postoperative mortality of hip fracture patients. As shown in Fig. 2A, the heterogeneity test (*I*^2^ = 69%, *P* = 0.0003) of the results of POSSUM's combined analysis of the morbidity indicates that the overall heterogeneity is high, so the random-effects model is used to combine the effect size, the result is RR = 1.07 (95% CI 0.93–1.24, *P* = 0.35). As shown in Figs. 2B and 3C, the heterogeneity test of the results of the combined analysis by POSSUM and P-POSSUM (*I*^2^ = 87%, *P* < 0.00001; *I*^2^ = 0%, *P* = 0.65) on the postoperative mortality suggesting that the heterogeneous of POSSUM to mortality is high, so the random effects model is used for POSSUM and the fixed effects model is used for P-POSSUM to combine the effect size, and the final results are RR = 1.93 (95% CI 1.21–3.08, *P* = 0.006) and RR = 1.15 (95% CI 0.89–1.50, *P* = 0.29), respectively.

### Results of sensitivity and subgroup analysis of POSSUM in predicting postoperative morbidity and mortality in patients with hip fractures

Sensitivity analysis was performed with RevMan5.4 software, we found that the study of Blay-Domínguez et al. [20] is a significant effect on the overall heterogeneity in POSSUM to morbidity. The prediction by POSSUM of mortality of patients with hip fractures was analyzed in subgroups according to the fracture type (femoral neck fractures, intertrochanteric fractures, and hip fractures) and sample size (sample > 200 cases, sample < 200 cases). As shown in Fig. 3A, after excluding studies that led to major sources of heterogeneity, RR = 1.01 (95% CI 0.94–1.08, *I*^2^ = 18%) for POSSUM to morbidity. In Fig. 3B, the RR of sample > 200 subgroup = 1.59 (95% CI 1.06–2.40, *I*^2^ = 83%) and sample < 200 subgroup = 2.45 (95% CI 0.71–8.42, *I*^2^ = 90%) for POSSUM to mortality. In Fig. 3C, the RR of intertrochanteric neck fracture subgroup = 5.04 (95% CI 1.07–23.75, *I*^2^ = 84%), RR of femoral femoral fracture subgroup = 1.43 (95% CI 1.10–1.84, *I*^2^ = 17%), RR of hip fracture subgroup = 1.69 (95% CI 0.87–3.32, *I*^2^ = 87%).

## Discussion

According to research reports that can be retrieved so far, this study is the first comprehensive analysis of the application of POSSUM and P-POSSUM scoring systems to elderly hip fractures. In terms of the results of this study, the POSSUM scoring system can accurately predicted the postoperative morbidity (RR = 1.07, 95% CI 0.93–1.24), and compared with POSSUM over-predicted the mortality (RR = 1.93, 95% CI 1.21–3.08), P-POSSUM can accurately predict the postoperative mortality of elderly patients with hip fractures (RR = 1.15, 95% CI 0.89–1.50).

In a meta-analysis report on the application value of POSSUM scoring system in hepatobiliary and pancreatic surgery by Chen et al. [33], they believe that because the early POSSUM scoring model is not suitable for current surgical operations, it led to POSSUM’s over-prediction of postoperative morbidity and mortality. Similarly, we also believe that the same situation may exist here. The POSSUM scoring system proposed by Copeland et al. in 1991 was originally used for general surgery patients. It was used in orthopedics after Mohamed et al. [18] modified its surgical scoring table according to the characteristics of orthopedic surgery in 2002. In the past 18 years, surgical methods and instruments have been greatly improved, and the nursing teams in related departments have become more professional. Previous improvements according to the characteristics of orthopedic surgery are no longer applicable to the current postoperative risk assessment. And, with the development of surgical technology, the impact of surgical intervention on the prognosis of patients is getting smaller and smaller. Combining the characteristics of high incidence of hip fracture in the elderly patients and having many underlying diseases in this group of people, it is not enough to modify the surgical severity score scale alone, and the physiological score scale should also be specifically modified. Orthopedic patients, especially those with hip fractures, have a tendency to stay in bed for a long time, which leads to a high risk of venous thrombosis. The physiology score scales, such as coagulation function, D-dimer, and other auxiliary examinations such as neck and lower extremity vascular color Doppler ultrasound to assess the patient's vascular condition, have not been reflected in the score.

Secondly, some studies reported that the difference in the prediction by POSSUM on the morbidity and mortality may be caused by the difference in the medical technology level, population characteristics, and the composition ratio of patient operation types in the non-originating country of the system from the originating country of the system [12]. In the process of exploring the source of heterogeneity of combined results in this study, we found that the type of hip fracture, the choice of surgical methods, and the distribution of patient age may all have an impact on the prediction accuracy of the POSSUM scoring system. Because the treatment methods of different fracture types are often different, the prognosis is naturally very different; and advanced surgical methods and young patients show a lower risk of postoperative death and complications. In addition, the POSSUM score of elderly patients may have false score values. For example, a study by Ramanathan et al. [21] found that elderly people older than 80 years old are abnormal compared to normal people because the relevant test results are "normal" for themselves, which leads to the inability of the POSSUM score to accurately predict the postoperative complications rate and mortality of this group of people. That is, POSSUM gives an excessively high score for the "normal" physiological examination results that can maintain the physiological function of this group of people. Therefore, this group population may not be suitable for POSSUM scoring system, a two-component scoring model of preoperative physiology score and surgical severity score. We believe that the above problems may be solved by risk grouping based on the size of the POSUUM score value. From previous reports, it can be seen that the error of POSSUM's over-predicting postoperative death events mainly comes from the low-risk group. For example, in the reports of Whiteley et al. [10] and Prythech et al. [34], The predicted value of death event in the low-risk group was 6 times and 7 times the actual observation value, and POSSUM performed well in the high-risk group. Therefore, we believe that it is necessary to improve the predictive ability of the POSSUM scoring system in the complication rate and mortality of patients with hip fractures, and it is very necessary to predict the risk of patients when using the scoring system.

It is worth raising that, according to previous reports in the literature, the overall prediction of the POSSUM scoring system for postoperative patients was consistent with actual observations, but its prediction for younger patients and patients undergoing elective surgery was found to be higher than the actual outcome in the subgroup [35], so this seems to suggest that the elderly population and critically ill patients requiring emergency surgery may be the priority groups. Evidence for this view is also provided by a recently retrospective cohort study, which showed that the P-POSSUM score showed good predictive power for postoperative mortality in COVID-19 positive patients undergoing emergency general surgery [36]. The scoring system is not applicable to children because the physiological scoring indicators in the scoring system are those of adults. In addition, the definition of postoperative complications is not completely clear, especially for those with preoperative underlying disease, so a clear definition of postoperative complications is necessary. Finally, according to the regulations of the POSSUM scoring system when it was established, the physiological indicators of the system should preferably be data within 1 day before surgery, and POSSUM is best for predicting complications and mortality within 30 days after surgery. And regarding the relevant data in the surgical scoring, such as blood loss and operative time, they need to be recorded accurately by the assessor.

One of the shortcomings of this study is that although our study was analysed in subgroups, we did not find a major source of heterogeneity in the POSSUM-predicted postoperative mortality pairs. We suspect that this may be due to differences in the cause of the fracture, the physical impact of the fracture itself, the treatment, the distribution of the population, and the level of medical care, but we were unable to draw firm conclusions. In addition, this study also failed to collect relevant unpublished data.

In all, the current data analysis shows that the POSSUM scoring system can predict the postoperative morbidity of elderly hip fracture patients. POSSUM's prediction of mortality is affected by the type of fracture and size of sample. Compared with POSSUM, P-POSSUM can accurately predict the postoperative mortality of patients with hip fractures. The application of the POSSUM scoring system in orthopedics needs to be further improved according to the characteristics of orthopedic patients and surgery, as well as needing more multi-center and large-sample prospective studies so that it can be more applicable to orthopedics.

## Declarations

### Research registration unique identifying number (UIN)

Name of registry: PROSPERO for systematic reviews and meta-analysis. Unique identifying number or registration ID: CRD42021234418. Hyperlink to the registration: https://www.crd.york.ac.uk/prospero
